# miRNA-Mediated Regulation of Ferroptosis in Neurological Disorders: Mechanisms and Therapeutic Implications

**DOI:** 10.3390/ijms27094037

**Published:** 2026-04-30

**Authors:** Chenyu Wang, Tingrui Luo, Nanhao Zhou, Xianbo Mou

**Affiliations:** Health Science Center, Ningbo University, Ningbo 315211, China; 15906611615@163.com (C.W.);

**Keywords:** miRNA, ferroptosis, neurological disorders, exosomes

## Abstract

Ferroptosis is a form of regulated cell death driven by iron-dependent phospholipid peroxidation and has emerged as a key mechanism of neuronal injury across a broad spectrum of neurological disorders. MicroRNAs (miRNAs), which function primarily as post-transcriptional regulators of gene expression, are increasingly recognized as important modulators of the regulatory networks governing ferroptosis and as potential therapeutic targets in these conditions. In this review, we synthesize current advances in miRNA-mediated regulation of ferroptosis in neurological disorders. We first outline the core molecular pathways governing ferroptosis, with particular emphasis on antioxidant defense, lipid peroxidation, and iron metabolism. We then integrate evidence from ischemic stroke, intracerebral hemorrhage, epilepsy, toxic encephalopathy, spinal cord injury, Parkinson’s disease, and Alzheimer’s disease, to illustrate how disease-specific miRNA regulatory axes shape ferroptotic vulnerability and its pathological consequences in distinct neurological settings. Importantly, we highlight exosome-based strategies targeting ferroptosis-related miRNA networks as a promising therapeutic approach for neurological disorders, with demonstrated neuroprotective and functional benefits in preclinical studies. Collectively, current evidence supports miRNA-mediated regulation of ferroptosis as an important mechanistic framework and a promising therapeutic target in neurological disorders.

## 1. Introduction

Neurological disorders characterized by irreversible neuronal injury impose a substantial medical and societal burden. In 2021, disorders affecting the nervous system affected 3.40 billion individuals worldwide and accounted for 443 million disability-adjusted life-years (DALYs), making them the leading cause of DALYs among all disease groups worldwide [1]. Within this broad disease spectrum, conditions involving acute or progressive neuronal loss are particularly devastating because mature neurons in the central nervous system have severely limited regenerative capacity. As a result, neuronal injury often leads to permanent functional deficits and persistent neurological disability [2]. This is particularly evident in ischemic stroke, intracerebral hemorrhage, epilepsy, spinal cord injury, toxic encephalopathy, Parkinson’s disease, and Alzheimer’s disease, in which neuronal damage contributes substantially to long-term cognitive, sensory, and motor dysfunction. Together, these features underscore the urgent need to identify pathogenic mechanisms that can be therapeutically targeted to achieve meaningful neuroprotection.

Among the regulated cell death pathways implicated in neurological injury, ferroptosis has emerged as an important pathogenic mechanism and a potential therapeutic target [3]. Ferroptosis is an iron-dependent form of regulated cell death characterized by the accumulation of membrane lipid peroxides and is closely associated with oxidative stress, disturbed iron homeostasis, and impaired antioxidant defense systems [4]. In the nervous system, where neuronal membranes are enriched in polyunsaturated fatty acid-containing phospholipids and redox homeostasis must be tightly maintained, these disturbances may render neurons particularly susceptible to ferroptotic damage [5]. Notably, dysregulated iron homeostasis is a recurrent pathological feature in multiple neurological disorders, particularly stroke and neurodegenerative diseases, further reinforcing the mechanistic link between ferroptosis and neurological injury [6]. These observations collectively underscore the pathogenic relevance of ferroptosis across multiple neurological disorders, yet effective and mechanism-based therapeutic strategies remain limited [7].

MicroRNAs (miRNAs) are key post-transcriptional regulators and are increasingly recognized as important modulators of the molecular pathways underlying ferroptosis [8]. Because individual miRNAs can coordinately regulate multiple target mRNAs, they are well positioned to influence the interconnected processes underlying ferroptosis, including iron homeostasis, lipid peroxidation, and antioxidant defense [8,9]. These regulatory effects can also be shaped by competing endogenous RNA (ceRNA) networks, in which certain long non-coding RNAs (lncRNAs) and circular RNAs (circRNAs) can act as molecular sponges for shared miRNAs, thereby modulating ferroptosis-related gene expression and cellular susceptibility to ferroptosis [10]. In addition, miRNA-mediated regulation may interact with DNA methylation-dependent epigenetic mechanisms in neurological disorders, with potential implications for ferroptosis-related neuronal injury [11,12]. Beyond these intracellular functions, miRNAs can also be packaged into exosomes, which protect them from nuclease degradation, facilitate intercellular transfer, and may enable delivery across the blood–brain barrier [13]. In the nervous system, exosomal miRNAs contribute to neuron–glia and brain–periphery communication and have attracted growing interest as minimally invasive biomarkers and potential therapeutic vehicles [13,14]. Exosome engineering further enhances their translational potential by providing a biocompatible platform for RNA delivery to the injured nervous system, although challenges in cargo loading, targeting specificity, large-scale production, and standardization remain [15]. However, despite accumulating evidence supporting a regulatory role for miRNAs in ferroptosis, their disease-specific roles across neurological disorders have not yet been systematically synthesized.

In this review, we summarize current advances in the miRNA-mediated regulation of ferroptosis in neurological disorders. We first outline the core molecular pathways underlying ferroptosis, with a focus on antioxidant defense, lipid peroxidation, and iron metabolism. We then examine how miRNAs regulate ferroptosis in representative neurological disorders, including ischemic stroke, intracerebral hemorrhage, epilepsy, toxic encephalopathy, spinal cord injury, Parkinson’s disease, and Alzheimer’s disease, and highlight emerging preclinical evidence supporting exosome-based strategies targeting ferroptosis-related miRNA networks as a potential therapeutic approach. Finally, we address the major translational challenges that must be overcome before miRNA-based ferroptosis-targeting strategies can be advanced toward clinical application.

## 2. Core Molecular Pathways Underlying Ferroptosis in Neurological Disorders

Ferroptosis is an iron-dependent form of regulated cell death characterized by phospholipid peroxidation in polyunsaturated fatty acid (PUFA)-containing membranes [4]. Neurons are particularly susceptible to ferroptotic injury because of their high metabolic rate and oxidative burden, their PUFA-enriched membrane composition, and tightly regulated iron homeostasis, features that together help explain the vulnerability of neural tissue across multiple neurological disorders [16,17]. Accordingly, the molecular basis of ferroptosis in the nervous system can be broadly organized into three interconnected modules: antioxidant defense, lipid peroxidation, and iron metabolism. These pathways collectively govern ferroptotic vulnerability and provide the mechanistic framework for interpreting disease-specific miRNA regulation in the following sections (Table 1).

### 2.1. Antioxidant Defense

The system x_c_^−^−glutathione (GSH)–glutathione peroxidase 4 (GPX4) axis is a pivotal antioxidant pathway governing ferroptosis in neurons. GSH serves as an essential cofactor for GPX4, which neutralizes lipid peroxides and limits oxidative damage. GSH availability depends on system x_c_^−^, a plasma membrane cystine/glutamate antiporter composed of solute carrier family 7 member 11 (SLC7A11) and solute carrier family 3 member 2 (SLC3A2) that imports extracellular cystine in exchange for intracellular glutamate, after which cystine is reduced to cysteine for GSH synthesis [30].

Nuclear factor erythroid 2-related factor 2 (Nrf2) is a master regulator of antioxidant defense that orchestrates the cellular response to oxidative stress. It binds to antioxidant response elements (AREs) in the promoter regions of cytoprotective and antioxidant genes [31]. Under basal conditions, Nrf2 is sequestered in the cytoplasm by Kelch-like ECH-associated protein 1 (Keap1), which promotes its ubiquitination and degradation [32]. Oxidative stress disrupts the Nrf2-Keap1 interaction, enabling Nrf2 to translocate into the nucleus, bind to AREs, and initiate transcription of downstream genes such as heme oxygenase 1 (*HMOX1*) and *GPX4* [31]. GPX4 directly reduces phospholipid hydroperoxides, whereas heme oxygenase 1 (HO-1) contributes to adaptive antioxidant responses but may exert context-dependent effects on ferroptosis because heme degradation can also increase intracellular free iron [33,34]. Additional regulators, such as gamma-aminobutyric acid receptor subunit beta-1 (GABPB1), may further modulate Nrf2 activity and ferroptotic susceptibility [35,36]. The Nrf2–Keap1 pathway has emerged as an important regulator of ferroptosis-related oxidative injury in neurological disorders, particularly in neurodegenerative diseases and ischemic stroke [37,38].

### 2.2. Lipid Peroxidation

Lipid peroxidation is the central biochemical event in ferroptosis, whereby iron-dependent oxidation of polyunsaturated fatty acid (PUFA)-containing phospholipids leads to the accumulation of lipid peroxides, disruption of membrane integrity, and ultimately cell death [4]. Neurons are especially vulnerable because of their high PUFA content, particularly long-chain PUFAs such as eicosapentaenoic acid (EPA; ω-3 PUFA) and arachidonic acid (AA; ω-6 PUFA), which are prone to oxidative modification [4,39]. The enzyme acyl-CoA synthetase long-chain family member 4 (ACSL4) plays a critical role in ferroptosis by activating free PUFAs through esterification with coenzyme A, thereby generating PUFA-CoA species [40]. Lysophosphatidylcholine acyltransferases (LPCATs), particularly LPCAT3, incorporate these PUFA-CoA derivatives into membrane phospholipids, sensitizing the membranes to peroxidation [41]. Together, ACSL4 and LPCATs expand the pool of peroxidation-prone membrane phospholipids, thereby increasing susceptibility to ferroptotic damage.

Lipid peroxidation can be catalyzed by enzymes such as lipoxygenases (ALOXs), although their role may have been overestimated in earlier studies [42,43]. Cytochrome P450 oxidoreductase (POR) has emerged as a critical contributor to lipid peroxidation, with the capacity to promote oxidation of PUFA-containing phospholipids within cellular membranes [42,43]. Knockdown studies have shown that *ACSL4* depletion is more protective against ferroptosis than *LPCAT3* silencing [44], underscoring ACSL4’s central role in this process.

### 2.3. Iron Metabolism

Iron metabolism is a core determinant of ferroptosis because intracellular iron accumulation fuels iron-dependent lipid peroxidation. Elevated levels of ferrous iron (Fe^2+^) promote the Fenton reaction, generating highly reactive hydroxyl radicals (OH·) that induce oxidative damage to cellular macromolecules and promote membrane lipid peroxidation [45]. This iron-dependent oxidative stress is a central driver of ferroptosis and has been increasingly implicated in the pathogenesis of various neurological disorders [46].

Cellular iron exists in two primary forms: the labile iron pool (LIP) and protein-bound iron [47]. The LIP represents a small but metabolically active pool of redox-active ferrous iron that participates in multiple biochemical reactions and serves as the primary source of Fe^2+^ for the Fenton reaction [48]. Although essential for normal cellular metabolism, excessive expansion of the LIP can dramatically enhance oxidative stress and ferroptosis susceptibility [49,50]. In contrast, protein-bound iron is involved in iron storage and transport, thereby maintaining cellular iron homeostasis [51]. Ferritin stores excess intracellular iron in its ferric form (Fe^3+^). It consists of ferritin heavy chain 1 (FTH1) and ferritin light chain (FTL) subunits, and each ferritin complex can store up to ~4500 iron atoms [52].

Iron transport in the circulation is primarily mediated by transferrin (Tf), a glycoprotein capable of binding two ferric (Fe^3+^) ions with high affinity [53]. Through its interaction with the transferrin receptor 1 (TfR1), Tf regulates cellular iron uptake and participates in systemic iron homeostasis [54].

Disruption of ferritin metabolism can alter the balance between free and stored iron and thereby promote ferroptosis [55]. Ferritin degradation occurs via ferritinophagy, a selective form of autophagy mediated by nuclear receptor coactivator 4 (NCOA4), which functions as a cargo receptor for ferritin [56]. Overexpression of NCOA4 accelerates ferritin degradation, increases the LIP, and enhances ferroptosis susceptibility [55]. These observations support the concept that altered ferritin turnover can directly modulate ferroptotic sensitivity.

Cellular iron metabolism involves tightly regulated processes of uptake, trafficking, storage, and export [57]. Transferrin binds to the TfR1 on the cell membrane and is internalized via endocytosis, where Fe^3+^ is reduced to Fe^2+^ by six-transmembrane epithelial antigen of prostate 3 (STEAP3) [58]. Fe^2+^ is subsequently transported to the cytosol through divalent metal transporter 1 (DMT1), contributing to the LIP [59]. Cytosolic iron can be utilized for metabolic processes, stored in ferritin, or exported through ferroportin (FPN), the only known iron exporter in mammalian cells [60]. Dysregulated FPN activity can lead to intracellular iron accumulation and enhance ferroptosis sensitivity [61,62]. In neurological disorders such as Alzheimer’s disease (AD), reduced FPN expression contributes to brain iron overload, thereby promoting ferroptosis-mediated neuronal damage and cognitive decline [63].

To provide a focused overview, Table 1 summarizes selected studies on key molecular regulators of ferroptosis and their regulatory miRNAs in neurological disorders. Studies were included based on three criteria: involvement of key molecular regulators of ferroptosis, relevance to neurological disorders, and identification of a clearly defined miRNA–target regulatory axis.

## 3. Disease-Specific MicroRNA Regulation of Ferroptosis in Neurological Disorders

### 3.1. Ischemic Stroke

Stroke remains a major global health challenge, ranking as the third leading cause of death and the fourth leading cause of DALYs worldwide in 2021 [64]. Approximately 70% of all stroke cases are ischemic, disproportionately affecting populations in low- and middle-income countries [65]. Ischemic stroke arises from cerebral infarction due to inadequate blood supply, initiating a cascade of pathological events, including excitotoxicity, oxidative stress, and neuronal death via membrane disruption and cell lysis [66]. Effective management focuses on revascularization to restore blood flow and mitigate secondary neuronal damage. Intravenous thrombolysis and endovascular thrombectomy (EVT) are currently the cornerstones of acute ischemic stroke treatment [67]. However, extended ischemia or ischemia/reperfusion (I/R) injuries often result in irreversible neuronal damage, contributing to complications such as cognitive impairments, epilepsy, depression, fatigue, tremors, dysphagia, and pain, all of which significantly diminish the quality of life and worsen outcomes [68]. Despite substantial advances in reperfusion therapy, effective interventions that directly attenuate post-stroke neuronal injury remain limited.

Preventing permanent neuronal loss after ischemic stroke requires an in-depth understanding of the underlying mechanisms of neuronal cell death and the identification of therapeutic targets. Accumulating evidence indicates that post-stroke neuronal injury is closely associated with ferroptosis, as evidenced by lipid peroxidation, iron accumulation, reduced GPX4 expression, increased ACSL4 and COX-2 expression, and elevated Reactive Oxygen Species (ROS) levels [69]. For instance, ACSL4 has been implicated in exacerbating ischemic stroke through ferroptosis-induced brain injury and neuroinflammation [70]. In parallel, hypoxia-inducible factor-1α (HIF-1α), a master regulator of the cellular hypoxic response, is increasingly recognized as a potential link between ischemic stroke and ferroptotic injury, as it modulates oxidative stress, iron homeostasis, and downstream effectors such as HO-1 within the ischemic microenvironment [71]. Together, these findings identify ferroptosis-related signaling as an important pathogenic component of ischemic stroke and provide a mechanistic basis for miRNA-targeted intervention.

**Figure 1:**
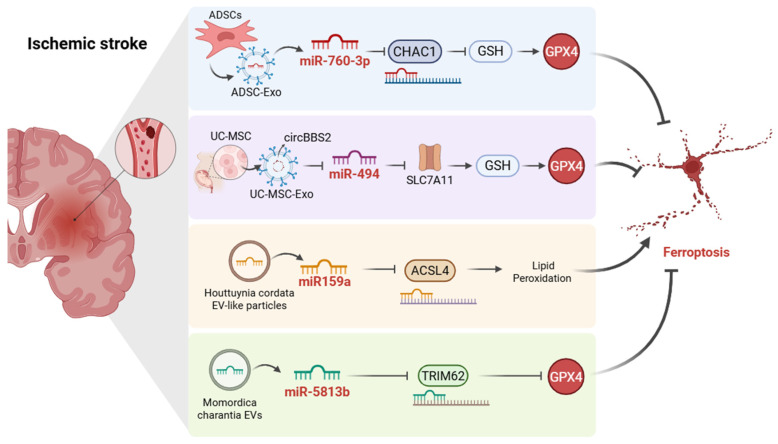
Mechanisms of microRNA-Mediated Regulation of Ferroptosis in Ischemic Stroke. adipose-derived stem cell (ADSC)-derived exosomal miR-760-3p downregulates ChaC glutathione-specific γ-glutamylcyclotransferase 1 (*CHAC1*), thereby preserving glutathione (GSH) homeostasis and supporting GPX4-dependent antioxidant defense. Umbilical cord mesenchymal stem cell (UC-MSC)-derived exosomal *circBBS2* suppresses miR-494, resulting in increased solute carrier family 7 member 11 (SLC7A11) expression and reinforcement of the SLC7A11/GSH/GPX4 axis. *Houttuynia cordata*-derived vesicle-like particles deliver miR-159a to inhibit acyl-CoA synthetase long-chain family member 4 (*ACSL4*) and reduce lipid peroxidation. *Momordica charantia*-derived extracellular vesicles deliver miR-5813b to inhibit tripartite motif containing 62 (*TRIM62*), thereby reducing GPX4 ubiquitination and stabilizing GPX4. Collectively, these regulatory events are associated with suppression of ferroptotic injury in ischemic stroke. Arrows indicate positive regulation or increase, whereas blunt-ended lines indicate inhibition or decrease. Created in BioRender. Wang, C. (2026) https://BioRender.com/d1kced1, accessed on 25 April 2026.

Against this background, miRNA-based strategies targeting ferroptosis have emerged as a promising approach to mitigate ischemic brain injury and improve neurological outcomes (Figure 1). Exosomes and other extracellular vesicles have attracted growing attention as therapeutic carriers in ischemic stroke because of their favorable biocompatibility, low immunogenicity, and potential to cross the blood–brain barrier (BBB) [72]. For example, intranasal administration of adipose-derived stem cell exosomes (ADSC-Exo) enriched with miR-760-3p has shown significant neuroprotective effects in middle cerebral artery occlusion (MCAO) models [73]. This treatment reduced neurological severity scores and enhanced neurobehavioral performance in mice. Mechanistically, miR-760-3p downregulated ChaC glutathione specific gamma-glutamylcyclotransferase 1 (*CHAC1*), a γ-glutamylcyclotransferase that degrades GSH. *CHAC1* suppression preserved GSH levels, thereby sustaining GPX4 function, restoring antioxidant defenses, and inhibiting ferroptosis [74]. Notably, the intranasal delivery method bypassed the BBB, reducing systemic side effects and enabling lower therapeutic doses [75].

Mesenchymal stem cell-based interventions have also shown promise in ischemic stroke, particularly through umbilical cord mesenchymal stem cell (UC-MSC)-derived exosomes [76,77]. A recent study demonstrated that UC-MSC-derived exosomes carrying *circBBS2* inhibited ferroptosis by sponging miR-494, thereby increasing SLC7A11 expression [19]. SLC7A11 enhances cystine import, a critical precursor for GSH synthesis, thus mitigating oxidative damage and alleviating ischemic stroke outcomes. These findings further support the therapeutic relevance of the SLC7A11/GSH/GPX4 axis in ischemic stroke.

Recent studies have shown that anti-ferroptotic small RNA delivery can also be achieved by non-mammalian vesicles, thereby broadening the therapeutic landscape. Extracellular vesicle-like particles derived from *Houttuynia cordata* Thunb. alleviated ischemic brain injury by delivering miR-159a, which directly targeted *ACSL4* and suppressed ferroptosis in middle cerebral artery occlusion (MCAO) mice [28]. In addition to reducing infarct volume and improving neurological function, these vesicle-like particles preserved BBB integrity and attenuated mitochondrial damage, highlighting the therapeutic relevance of the miR-159a/*ACSL4* axis in ischemic stroke [28]. Similarly, small extracellular vesicles derived from *Momordica charantia* mitigated neuronal ferroptosis after ischemic stroke through delivery of miR-5813b, which targeted tripartite motif containing 62 (*TRIM62*) and thereby inhibited GPX4 ubiquitination, stabilizing GPX4 and attenuating ferroptotic injury [78]. These findings provide mechanistic insight into the anti-stroke effects of traditional herbal medicines from the perspective of miRNA-mediated ferroptosis regulation.

Overall, these studies indicate that miRNA-mediated regulatory networks modulate ferroptosis in ischemic stroke through complementary mechanisms, including regulation of redox homeostasis and lipid peroxidation. This evolving body of work provides a more integrated mechanistic view of ferroptosis in ischemic stroke and suggests several candidate targets for anti-ferroptotic intervention. However, the available evidence remains largely preclinical, and major challenges related to delivery efficiency, cargo heterogeneity, dose standardization, and clinical translatability still need to be addressed.

### 3.2. Intracerebral Hemorrhage

Intracerebral hemorrhage (ICH) is a devastating subtype of stroke with high morbidity and mortality rates [19,79]. Although ICH accounts for only approximately 15% of all stroke cases, it is responsible for nearly 50% of stroke-related deaths and contributes substantially to long-term disability [79]. ICH is defined as bleeding into the brain parenchyma caused by rupture of a cerebral blood vessel and most commonly arises from hypertensive small-vessel disease or cerebral amyloid angiopathy [80]. Current therapeutic approaches prioritize early blood pressure management, minimizing hematoma expansion, and optimizing surgical interventions [81]. However, effective therapies targeting secondary brain injury remain limited, largely because the mechanisms driving post-hemorrhagic neuronal damage are still incompletely understood.

**Figure 2:**
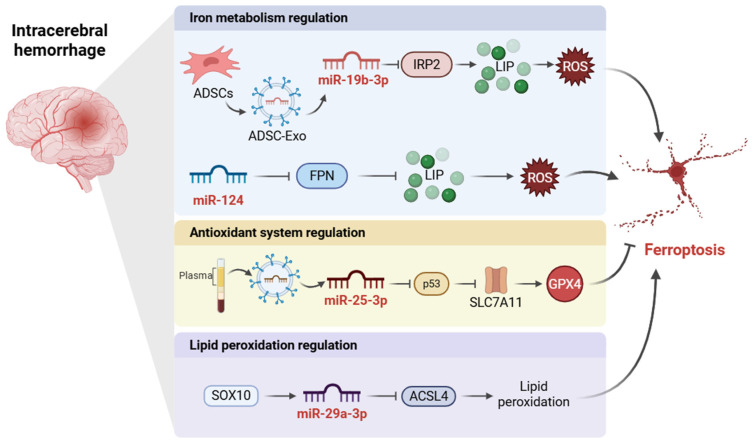
Mechanisms of miRNA-Mediated Regulation of Ferroptosis in Intracerebral Hemorrhage. Adipose-derived stem cell (ADSC)-derived exosomal miR-19b-3p downregulates iron regulatory protein 2 (IRP2), thereby reducing iron accumulation and oxidative stress. In contrast, miR-124 suppresses ferroportin (FPN), contributing to intracellular iron retention and ferroptosis-associated injury. Exosomal miR-25-3p attenuates ferroptotic injury through the p53/solute carrier family 7 member 11 (SLC7A11)/glutathione peroxidase 4 (GPX4) axis. In addition, SRY-box transcription factor 10 (SOX10)-induced miR-29a-3p inhibits acyl-CoA synthetase long-chain family member 4 (*ACSL4*), reduces lipid peroxidation, and mitigates ferroptotic damage. Collectively, these pathways highlight the importance of iron handling, redox homeostasis, and lipid peroxidation in miRNA-mediated regulation of ferroptosis after intracerebral hemorrhage. Arrows indicate positive regulation or increase, whereas blunt-ended lines indicate inhibition or decrease. Created in BioRender. Wang, C. (2026) https://BioRender.com/kvlgqwh, accessed on 25 April 2026.

Increasing evidence identifies ferroptosis as an important contributor to secondary brain injury after ICH. Following hemorrhage, erythrocyte lysis releases hemoglobin-derived iron into the brain parenchyma, thereby driving iron overload, oxidative stress, lipid peroxidation, and neuronal death [82]. Moreover, disruption of iron homeostasis may further exacerbate ferroptotic injury after ICH through heme degradation-driven iron release, insufficient ferritin buffering, and increased neuronal iron influx via TfR1-dependent and calcium channel-mediated pathways [82]. Although iron chelation–based ferroptosis-targeting strategies have shown therapeutic promise, their clinical translation remains constrained by uncertain blood–brain barrier penetration, systemic toxicity, short half-life, and delivery-related limitations [83]. In this context, miRNA-mediated regulation of post-ICH ferroptosis has emerged as a promising area of investigation with both mechanistic and therapeutic relevance (Figure 2).

Exosome-mediated miRNA delivery has emerged as a promising strategy for attenuating ferroptotic injury after ICH. In a murine ICH model, exosomes derived from young healthy human plasma accumulated in the perihematomal region and improved neurological recovery [84]. Mechanistically, exosomal miR-25-3p attenuated ferroptotic injury through the p53/SLC7A11/GPX4 axis, thereby reducing neuronal damage and improving behavioral outcomes [84]. Similarly, exosomes derived from miR-19b-3p-modified adipose-derived stem cells (ADSCs) were shown to inhibit ferroptosis and improve neurological function after ICH by targeting iron regulatory protein 2 (IRP2), thereby reducing iron accumulation and oxidative injury [85]. These findings underscore the potential of exosome-based therapies as innovative approaches to mitigate iron-induced neuronal damage and enhance recovery in ICH patients.

In parallel, non-exosomal miRNA pathways have further clarified the molecular basis of ferroptotic injury after ICH. The miR-124/FPN axis has been implicated in aging-associated susceptibility to post-ICH iron dyshomeostasis and neuronal death [25]. Mechanistically, miR-124 negatively regulated FPN, whereas inhibition of miR-124 increased FPN expression, reduced iron accumulation, and attenuated neuronal injury in aged ICH models [25]. These findings underscore the importance of iron export pathways in the miRNA-mediated regulation of ferroptotic damage after ICH. In addition, mechanistic evidence has linked ferroptosis in ICH to the miR-29a-3p/*ACSL4* axis. A recent study showed that SRY-box transcription factor 10 (SOX10) directly promoted miR-29a-3p expression by binding to its promoter, thereby suppressing *ACSL4* transcription and implicating the SOX10/miR-29a-3p/*ACSL4* axis in ferroptosis-related neuronal injury after ICH [29].

Overall, current evidence indicates that miRNA-mediated regulation of ferroptosis in ICH converges primarily on three interconnected processes: iron handling, redox homeostasis, and lipid peroxidation. Together, these findings refine the mechanistic framework linking ferroptosis to secondary brain injury after ICH and highlight several candidate targets for anti-ferroptotic intervention. Nevertheless, the available evidence remains largely preclinical, and major challenges related to delivery efficiency, cargo heterogeneity, dose standardization, and clinical translatability must be overcome before these strategies can be advanced toward therapeutic application.

### 3.3. Epilepsy

Epilepsy is a chronic neurological disorder that imposed a substantial global health burden in 2021, affecting an estimated 51.7 million people worldwide, with over 80% of prevalent cases and epilepsy-related DALYs occurring in low- and middle-income countries [1]. It is characterized by recurrent unprovoked seizures caused by abnormal, excessive, or synchronous neuronal activity in the brain and is associated with diverse etiologies, including structural, genetic, infectious, metabolic, immune, and unknown causes [86,87]. Advances in epilepsy research have focused on mechanisms involving tau protein, amyloid precursor protein, iron deposition, neurotransmitters, and T-type calcium channels [88]. Surgical treatment for drug-resistant focal epilepsy has demonstrated significant efficacy, particularly in conditions such as focal cortical dysplasia (FCD), status epilepticus, and epilepsy in young children [89].

Among the regulated cell death pathways implicated in epilepsy, ferroptosis has attracted increasing attention because seizure-associated oxidative stress promotes lipid peroxidation and neuronal injury [90]. Iron overload, which is commonly observed in post-hemorrhagic and post-traumatic epilepsy, may further exacerbate seizure activity and neuronal damage [91]. Ferroptosis is increasingly recognized as a mechanistic contributor to epilepsy, promoting seizure-associated neuronal loss and cognitive impairment, while astrocyte activation may further amplify ferroptosis-related neuronal injury [92]. Pharmacological inhibition of ferroptosis has shown promise in experimental epilepsy models, with representative agents such as ferrostatin-1 and several natural compounds demonstrating anti-seizure and neuroprotective effects [92]. Consistent with this concept, dihydroartemisinin was recently reported to mitigate seizures and neuronal injury in epileptic mice by inhibiting ferroptosis through the sirtuin 1 (SIRT1)/forkhead box O1 (FOXO1)/SLC7A11/GPX4 axis, further supporting ferroptosis-targeting strategies as a promising therapeutic direction in epilepsy [93].

**Figure 3:**
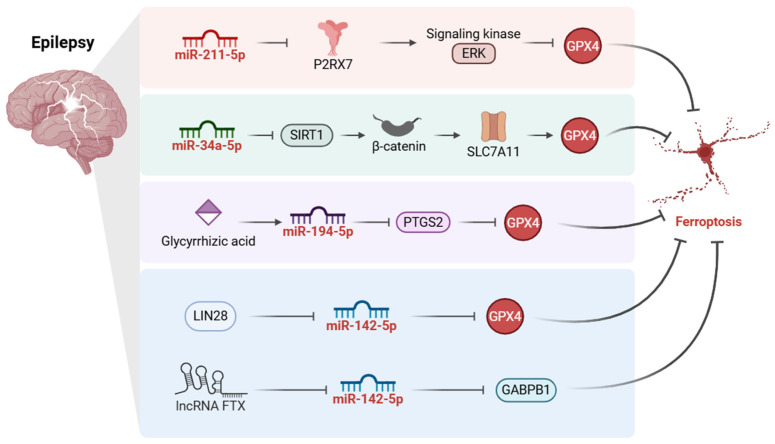
Mechanisms of miRNA-Mediated Regulation of Ferroptosis in Epilepsy. In epileptic models, decreased miR-211-5p is associated with increased P2X7 receptor (*P2RX7*) expression, extracellular signal-regulated kinase (ERK) activation, reduced GPX4, and enhanced ferroptotic vulnerability. Increased miR-34a-5p promotes ferroptosis by repressing sirtuin 1 (*SIRT1*) and modulating Wnt/β-catenin signaling. Glycyrrhizic acid exerts anti-ferroptotic effects through the miR-194-5p/prostaglandin-endoperoxide synthase 2 (*PTGS2*) axis. In addition, decreased *LIN28* is associated with increased miR-142-5p and reduced glutathione peroxidase activity and GPX4 expression, whereas long non-coding RNA (lncRNA) *FTX* suppresses miR-142-5p, thereby alleviating ferroptotic injury through the GA-binding protein transcription factor subunit beta 1 (*GABPB1*)-associated pathway. Collectively, these regulatory interactions support a mechanistic link between miRNA-centered networks and ferroptosis-related neuronal injury in epilepsy. Arrows indicate positive regulation or increase, whereas blunt-ended lines indicate inhibition or decrease. Created in BioRender. Wang, C. (2026) https://BioRender.com/4f3axqa, accessed on 25 April 2026.

Recent studies have begun to define specific RNA-mediated regulatory nodes linking ferroptosis to epileptogenesis (Figure 3). The miR-211-5p/P2X7 receptor (*P2RX7*)/extracellular signal-regulated kinase (ERK)/GPX4 axis has emerged as a representative pathway, in which downregulation of miR-211-5p in kainic acid-induced epilepsy is associated with increased *P2RX7* expression, enhanced oxidative stress, and ferroptosis-related neuronal injury, whereas restoration of miR-211-5p or silencing of *P2RX7* reduces seizure severity and duration [22]. Mechanistically, as an important danger sensor, P2RX7 detects extracellular nucleotides released from injured cells and promotes ERK-dependent signaling, which is linked to altered GPX4 levels and increased ferroptotic vulnerability in epilepsy [22,94]. MiR-34a-5p has recently been identified as another direct miRNA-mediated regulator of ferroptosis in epilepsy, being upregulated in the hippocampus of epileptic rats and in Mg^2+^-free hippocampal neuronal cultures, where it promotes neuronal ferroptosis by repressing *SIRT1* and thereby modulating the Wnt/β-catenin pathway [95].

Additional studies have expanded this regulatory network to include natural compounds and other ncRNAs [96]. Glycyrrhizic acid (GA) was shown to inhibit neuronal ferroptosis in a rat model of temporal lobe epilepsy (TLE) by modulating the miR-194-5p/*PTGS2* axis, thereby reducing seizure frequency and duration [97]. Hippocampal sclerosis (HS), characterized by neuronal loss and gliosis, is a common pathological feature of mesial temporal lobe epilepsy (MTLE) and related epilepsy syndromes [98]. In Mg^2+^-free in vitro models, hippocampal neurons exhibited decreased *LIN28* expression, increased miR-142-5p expression, reduced glutathione peroxidase activity and GPX4 expression, and elevated ROS and MDA levels, whereas *LIN28* overexpression reversed these pathological changes [99]. Consistent with this regulatory node, lncRNA *FTX* was shown to attenuate neuronal ferroptosis through the miR-142-5p/*GABPB1* axis [36]. Collectively, these findings further support the notion that miRNA-centered regulatory networks, modulated by both natural compounds and upstream ncRNAs, play an important role in epilepsy-associated ferroptosis and may influence both seizure severity and ferroptotic neuronal injury.

Overall, current evidence identifies ferroptosis as an important pathogenic mechanism in epilepsy and highlights miRNAs as key regulators of epilepsy-associated ferroptotic signaling. In particular, pathways such as the miR-211-5p/*P2RX7*/ERK/GPX4, miR-34a-5p/*SIRT1*, miR-194-5p/*PTGS2*, and miR-142-5p-related axes further refine the mechanistic framework linking ferroptosis to seizure-associated neuronal injury and cognitive impairment. Collectively, these findings support miRNA-centered ferroptotic pathways as mechanistically relevant and potentially actionable targets in epilepsy.

### 3.4. Toxic Encephalopathy

Multiple neurotoxic substrates in the environment could cause encephalopathy. However, their detailed mechanisms remain mysterious. Rapid development in the fields of regulated cell death and non-coding RNAs has established possible pathways. Evidence suggests that ferroptosis plays a significant role in the toxicity associated with exposure to metals such as arsenic, manganese, cadmium, nickel, aluminum, mercury, and lead through mechanisms involving disruption of iron metabolism, increased oxidative stress, and enhanced lipid peroxidation [100].

Lead exposure remains a severe public issue worldwide. It is associated with cognitive impairments, executive function alterations, social behavior abnormalities, and fine motor control deficits [101]. The direct neurotoxic effects of lead on the brain include apoptosis, excitotoxicity, mitochondrial damage, and other cellular disruptions, which may lead to permanent neurological damage [102]. Childhood exposure to lead can result in intellectual and learning disabilities as well as behavioral disorders, with these effects potentially persisting into adulthood [101]. Therefore, there is an urgent need to develop novel strategies for neuroprotection and neurorehabilitation in lead-induced encephalopathy. A recent study suggests that ferroptosis plays a role in lead-induced neurotoxicity in both the HT22 neuronal cell line and a mouse model, as evidenced by ferroptosis biomarkers such as the accumulation of Fe^2+^, increased levels of malondialdehyde (MDA), and decreased levels of GSH [20]. The study also attached importance to miRNA-mediated regulation. After screening miRNAs regulating *SLC7A11* using the TargetScan software and validating the results through dual-luciferase reporter assays, the researchers confirmed that miR-378a-3p directly binds to the 3′-UTR of *SLC7A11* [20]. Subsequently, by transfecting a miR-378a-3p inhibitor to suppress its expression, the researchers observed an increase in *SLC7A11* mRNA levels, a reduction in lipid ROS levels, and an elevation in GSH levels, indicating that inhibition of miR-378a-3p alleviates lead-induced ferroptosis [20]. These experiments demonstrate the association between lead exposure and ferroptosis and identify miR-378a-3p as a potential therapeutic target.

Manganese is an essential trace element for brain development and function; however, environmental exposure may lead to its accumulation in the basal ganglia, resulting in Parkinson’s disease-like symptoms [103]. The neurotoxic mechanisms of manganese include intracellular damage caused by oxidative stress, inflammation, excitotoxicity, and mitophagy [104]. It was recently reported in research that manganese exposure in the C57BL/6 mouse model causes anxiety-like behavior, according to the outcome of the Elevated Plus-Maze Test and Open Field Test [24]. Further mechanistic investigation demonstrates that Manganese exposure downregulates the expression of miR-125b-2-3p, while overexpression of miR-125b-2-3p mitigates manganese-induced ferroptosis by targeting *TfRC* [24]. This research complemented the neurological effect of manganese neurotoxicity and revealed a key pathway of miRNA-mediated regulation in toxin-induced neuronal ferroptosis.

Arsenic exposure poses a serious global health threat, affecting over 200 million people worldwide and contributing to a wide range of diseases, including skin conditions, cancer, cardiovascular diseases, diabetes, and neurological disorders [105]. Current knowledge about the mechanisms of arsenic toxicity involves multiple mechanisms, including oxidative stress, genotoxicity, and epigenetic modifications, which collectively lead to cellular damage, genetic alterations, and disrupted gene expression [106]. A recent study demonstrated that arsenic exposure caused learning and memory deficits in mice, with ferroptosis playing a crucial role in these impairments [107]. miR-21 was found to regulate ferroptosis in neurons by targeting *FTH1*, and its silencing reduced arsenic-induced ferroptosis and neuronal damage [107]. Additionally, exosomes derived from arsenic-exposed microglia carried miR-21, which further contributed to ferroptosis in neurons, highlighting the importance of intercellular communication in arsenic-induced neurotoxicity and suggesting miR-21 as a potential therapeutic target for mitigating arsenic-induced neurodegeneration [107].

In conclusion, the growing evidence linking ferroptosis to metal-induced neurotoxicity underscores the importance of understanding its mechanisms in toxic encephalopathy. Lead, manganese, and other metals disrupt cellular processes such as oxidative stress, mitochondrial dysfunction, and excitotoxicity, triggering ferroptotic neuronal death. The involvement of miRNAs in regulating ferroptosis pathways offers potential therapeutic targets, as exemplified by miR-378a-3p and miR-125b-2-3p. These findings emphasize the need for further research into miRNA-mediated neuroprotection strategies, offering new prospects for preventing or treating metal-induced encephalopathy and related neurological disorders.

### 3.5. Spinal Cord Injury

Spinal cord injury (SCI) is a complex pathological process triggered by acute trauma, causing multimolecular interactions in multiple cells, which lead to ischemia, inflammation, oxidative stress, and other destructive events [108]. Recovery mechanisms that include neuroprotection, immune modulation, and neuroregeneration show promise, but a definitive cure remains elusive, highlighting the need for further exploration into multifaceted therapeutic strategies to enhance treatment outcomes [108]. A recent review underscores the promising therapeutic potential of novel ferroptosis-inhibiting strategies, including mesenchymal stem cells (MSCs), extracellular vesicles (EVs), and transcranial magnetic stimulation (TMS), in treating SCI, suggesting that combining these approaches may alleviate neurological dysfunction by targeting ferroptosis [109]. These findings underscore the need for further investigation to develop targeted therapies. Collectively, current preclinical evidence indicates that novel miRNA-based therapeutics targeting ferroptosis show considerable potential for the treatment of SCI in experimental models.

Researchers started by discovering gene targets. A recent study identified the top ten ferroptosis-related genes through bioinformatics analysis. It confirmed increased mRNA expression of *STAT3*, *JUN*, *TLR4*, *ATF3*, *HMOX1*, *PTGS2*, and *RELA*, together with decreased mRNA expression of *VEGFA*, *MAPK1*, and *MAPK9* in a rat model of T10 spinal cord injury [110]. Through bioinformatics analysis, another study identified 41 differentially expressed ferroptosis-related genes (DE-FRGs) associated with acute SCI. It validated key DE-FRGs in blood samples from SCI patients and healthy controls [111]. Both studies built up complex lncRNA-miRNA-mRNA regulatory networks and predicted potential compounds that may target ferroptosis to repair SCI, providing new insights into the relationship between SCI and ferroptosis, highlighting potential therapeutic targets [110].

**Figure 4:**
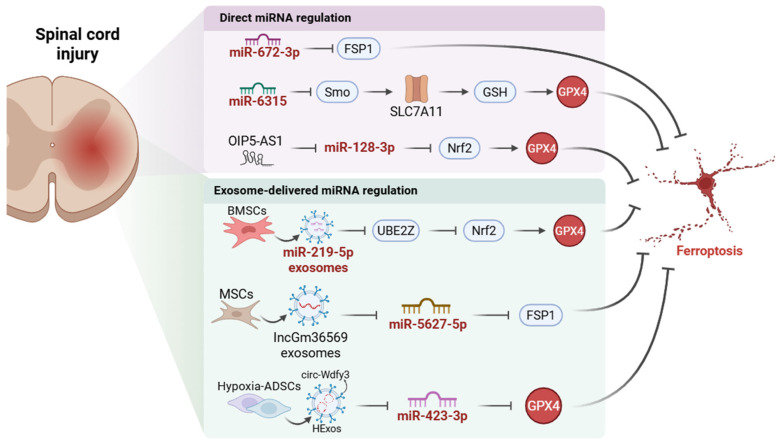
Mechanisms of miRNA-Mediated Regulation of Ferroptosis in Spinal Cord Injury. In direct miRNA regulation, miR-672-3p suppresses ferroptosis suppressor protein 1 (FSP1), thereby promoting ferroptosis. Silencing miR-6315 relieves repression of Smoothened (Smo) and is associated with restoration of solute carrier family 7 member 11 (SLC7A11), glutathione (GSH), and glutathione peroxidase 4 (GPX4), thereby attenuating ferroptosis. *OIP5-AS1* suppresses miR-128-3p, resulting in activation of the nuclear factor erythroid 2-related factor 2 (Nrf2)/GPX4 axis and inhibition of ferroptosis. In exosome-delivered non-coding RNA (ncRNA) regulation, bone marrow mesenchymal stem cell (BMSC)-derived exosomal miR-219-5p inhibits ubiquitin-conjugating enzyme E2 Z (*UBE2Z*) and activates the Nrf2-associated anti-ferroptotic pathway. Mesenchymal stem cell (MSC)-derived exosomal *lncGm36569* suppresses miR-5627-5p, thereby increasing FSP1 and inhibiting ferroptosis. Hypoxia-preconditioned adipose-derived stem cell (ADSC)-derived exosomes carrying *circ-Wdfy3* suppress miR-423-3p, resulting in increased GPX4 expression and reduced ferroptotic injury. Collectively, these findings support ncRNA-mediated ferroptosis regulation as a potential therapeutic axis in spinal cord injury. Arrows indicate positive regulation or increase, whereas blunt-ended lines indicate inhibition or decrease. Created in BioRender. Wang, C. (2026) https://BioRender.com/u97cgan, accessed on 25 April 2026.

Recent studies have underscored the pivotal role of miRNA-mediated ferroptosis regulation in spinal cord injury (SCI) treatment (Figure 4). In a rat model of contusive SCI, inhibition of miR-672-3p attenuated ferroptosis and improved functional recovery by restoring ferroptosis suppressor protein 1 (FSP1) expression [26]. This finding highlights the therapeutic potential of targeting miRNA pathways to reduce neuronal death and promote recovery. In another study, the regulatory function of lncRNA *OIP5-AS1* in SCI is explored, revealing its capacity to modulate ferroptosis via the miR-128-3p/Nrf2 axis [21]. Overexpression of *OIP5-AS1* enhances SCI recovery by inhibiting neuronal apoptosis and ferroptosis, further solidifying its therapeutic potential [21]. Additionally, a recent experimental study explores the role of miR-6315 in regulating ferroptosis post-SCI, demonstrating that silencing miR-6315 promotes functional recovery, neuronal axon regeneration, and reduced apoptosis in SCI rat models [112]. The findings suggest that miR-6315 modulates SCI repair by interacting with Smoothened (Smo) and ferroptosis-related factors such as SLC7A11, GSH, and GPX4 [112]. Taken together, the findings from these studies suggest that miRNA-mediated regulation of ferroptosis represents a promising strategy for SCI therapy. However, translating these findings into clinical applications will require addressing challenges related to delivery mechanisms and the specificity of miRNA targeting.

In recent studies, exosome-derived posttranscriptional regulation of ferroptosis has emerged as a promising therapeutic strategy for SCI, highlighting the potential of stem cell-derived exosomes as a novel miRNA-based treatment modality. Here, we introduce three recent investigations into this field. The first study demonstrates that miR-219-5p-rich exosomes derived from bone marrow mesenchymal stem cells (BMSCs) alleviate neuronal ferroptosis in SCI by targeting the *UBE2Z*/Nrf2 pathway [113]. The second study investigates the role of MSC-derived exosomes in acute SCI (ASCI), where *lncGm36569*-loaded exosomes inhibit neuronal ferroptosis via the miR-5627-5p/FSP1 axis, thus improving neuronal survival and promoting functional recovery. MSCs-exo treatment has shown significantly greater therapeutic effects in an ASCI mouse model compared to MSC transplantation alone, according to the results of the Basso Mouse Scale and Beam Walk Test [27]. This study emphasizes the therapeutic potential of MSC-exosomes in modulating iron overload and oxidative stress, which are key contributors to SCI pathology. The third study reveals that hypoxia-preconditioned adipose-derived stem cell (ADSC)-derived exosomes (HExos) significantly enhance SCI repair by modulating ferroptosis, specifically through the *circ-Wdfy3*-miR-423-3p-GPX4 pathway [18]. This research underscores the pivotal role of *circ-Wdfy3* in improving SCI outcomes by reducing oxidative stress and inflammation. These studies provide profound insights into the potential of exosome-based therapies in SCI treatment, positioning miRNA-driven ferroptosis regulation as a central mechanism for neuroprotection and repair. By leveraging the natural cargo of exosomes containing different regulatory ncRNAs, we can modulate iron homeostasis, mitigate oxidative damage, and consequently improve the outcome of SCI in animal models. However, the precise mechanisms underlying the differential effects of specific miRNAs and their delivery through exosomes require further elucidation, paving the way for more refined and personalized therapeutic strategies in SCI.

In conclusion, miRNA-mediated regulation of ferroptosis holds significant promise for treating SCI. Recent studies have demonstrated ncRNA-rich exosomes’ therapeutic potential and ability to modulate ferroptosis, promote neuronal survival, and enhance functional recovery in SCI models. These findings underscore the importance of targeting ferroptosis pathways, but the clinical application will require overcoming challenges in miRNA delivery and specificity. Future research should focus on refining these strategies for personalized, effective SCI treatments.

### 3.6. Parkinson’s Disease

Parkinson’s disease (PD) is a progressive neurodegenerative disorder marked by motor symptoms such as bradykinesia, resting tremor, rigidity, and postural instability, as well as non-motor manifestations including cognitive decline, affective disturbances, and autonomic dysfunction [114]. Pathologically, PD is characterized by the depletion of pigmented neurons and neuronal loss in the substantia nigra and locus coeruleus, along with the presence of Lewy bodies—eosinophilic cytoplasmic inclusions containing α-synuclein [115].

Ferroptosis, a form of regulated cell death driven by iron-dependent lipid peroxidation, plays a critical role in the pathogenesis of PD. This process has been observed in both cellular and animal models of PD, where ferroptosis inhibitors have shown promise in rescuing dopaminergic neurons from cell death [116]. Ferroptosis shares several pathological features with PD, including iron overload, lipid peroxidation, decreased GSH levels, reduced SLC7A11 expression, reduced DJ-1 levels, and reduced CoQ10 levels [117]. Additionally, α-synuclein aggregation drives ferroptosis by interacting with membranes, leading to calcium signaling abnormalities, lipid peroxidation, and iron accumulation. Clinical trials have explored the potential of ferroptosis inhibitors such as Deferiprone, N-acetylcysteine, and Coenzyme Q10, which have demonstrated varying degrees of therapeutic benefit, including improvements in motor symptoms, reductions in iron accumulation in the substantia nigra, and enhanced antioxidant capacity [118].

A recent bioinformatics study predicted several miRNAs, including miR-214/761/3619-5p, miR-203, miR-204/204b/211, miR-128/128ab, and miR-199ab-5p, as potential biomarkers and therapeutic targets for PD by regulating ferroptosis-related genes, according to miRCode [119]. As miRNA-based ferroptosis inhibitors continue to be developed, it is essential to review and evaluate recent discoveries in this field.

One such study investigated the role of miR-335 in ferroptosis in PD, focusing on its effect on *FTH1*. The researchers found that miR-335 is upregulated in PD models, leading to reduced *FTH1* expression, increased iron release, and exacerbated ferroptotic cell death. These effects were evidenced by decreased GPX4 and tyrosine hydroxylase (TH) levels. The study demonstrated that miR-335 directly targets *FTH1* mRNA, promoting lipid peroxidation, ROS accumulation, and mitochondrial dysfunction, thereby contributing to neurodegeneration. These findings suggest that miR-335 could be a key regulator of ferroptosis in PD, offering a potential therapeutic approach to modulate its effects on *FTH1* and acting as a biomarker for ferroptosis-related neurodegeneration [23].

A separate study explored the role of long non-coding RNA (lncRNA) *NEAT1* in ferroptosis, focusing on its modulation of the miR-150-5p/BRCA1-associated protein 1 (BAP1) signaling axis. The study demonstrated that *NEAT1* is upregulated in PD cell models, and its knockdown significantly improved cell viability by restoring antioxidant capacity (increased GSH levels) and reducing ROS, MDA, and iron accumulation. *NEAT1* functions as a competing endogenous RNA (ceRNA), binding miR-150-5p and modulating the miR-150-5p/BAP1/SLC7A11 axis, thus mitigating ferroptosis. These results highlight *NEAT1* as a key regulator of ferroptosis in PD and suggest its potential as a therapeutic target to mitigate ferroptosis-related neurodegeneration [120].

Another study examined the role of mammalian sterile 20-like kinase 1 (MST1) in ferroptosis regulation in a 1-methyl-4-phenylpyridinium (MPP+)-induced PD cell model. The study found that MST1 expression was elevated in PD, promoting apoptosis and autophagy. MiR-23b-3p was identified as a negative regulator of MST1, with its overexpression reducing ferroptosis, cell damage, and oxidative stress in PD cells. Inhibition of MST1 showed protective effects, though ferroptosis activation reversed this protection. These findings suggest that MST1 and miR-23b-3p may serve as potential therapeutic targets to mitigate ferroptosis in PD, though further animal and clinical validation is required [121].

Given the therapeutic strategies currently available for PD, such as dopamine supplementation, it is crucial to consider the receptor-related side effects that may arise from long-term dopamine use. Therefore, combining dopamine therapy with novel treatments targeting ferroptosis may hold promise in reducing neurodegeneration and improving patient outcomes.

### 3.7. Alzheimer’s Disease

Alzheimer’s disease (AD), a chronic and progressive neurodegenerative disorder and the leading cause of dementia, poses a substantial and growing global health challenge [122]. Although amyloid-β (Aβ) deposition and tau pathology remain the canonical neuropathological hallmarks of AD, accumulating evidence indicates that these pathological features are closely associated with iron dyshomeostasis, oxidative stress, and lipid peroxidation, suggesting a mechanistic link between the canonical pathological features of AD and ferroptosis-associated neuronal injury [123,124].

Several lines of evidence have strengthened the link between AD and ferroptosis. FPN, the only known mammalian non-heme iron exporter, is reduced in both APPswe/PS1dE9 mice and human AD brains [63]. Notably, neuron-specific FPN deficiency induces hippocampal atrophy, memory impairment, and ferroptotic injury, whereas restoration of FPN or pharmacological inhibition of ferroptosis attenuates neuronal loss and cognitive decline [63]. Familial AD-associated presenilin mutations further increase cellular susceptibility to ferroptosis by impairing selenium uptake and suppressing GPX4 expression, thereby linking genetically defined AD to defective anti-ferroptotic defense [125]. In parallel, nicotinamide adenine dinucleotide phosphate oxidase 4 (NOX4) has been identified as a key mediator of astrocytic ferroptosis in AD, where it promotes oxidative stress, lipid peroxidation, and mitochondrial metabolic dysfunction [126]. Together, these studies establish disturbed iron export, impaired GPX4-centered antioxidant defense, and oxidative stress as major determinants of ferroptosis susceptibility in AD.

Direct evidence for miRNA-mediated regulation of ferroptosis in AD has recently begun to emerge. A representative study identified the miR-7a/Krüppel-like factor 4 (*Klf4*) axis as a regulatory node linking ferroptosis to neuroinflammation in AD [127]. In APP/PSEN1 mouse brains and complementary cell-based models, elevated miR-7a targeted *Klf4* and modulation of this axis reduced labile iron accumulation, restored GPX4 expression and Nrf2-associated antioxidant signaling, and attenuated neuroinflammatory responses [127]. Importantly, this study provided functional evidence that a defined miRNA–target axis can simultaneously modulate ferroptotic and inflammatory phenotypes in an AD context [127].

More recently, this regulatory framework has been extended to astrocyte–neuron interactions. In APP/PS1 mice and primary astrocytes, Diminazene upregulated miR-10b-3p, which directly targeted *NOX4* and thereby suppressed astrocytic oxidative stress and inflammatory signaling [128]. Inhibition of miR-10b-3p substantially reversed these effects, and conditioned-medium experiments further indicated that modulation of the miR-10b-3p/*NOX4* axis attenuated neuronal ferroptosis downstream of astrocytic activation [128].

At the therapeutic level, a recent preclinical study showed that miRNA34-loaded bovine milk-derived small extracellular vesicles mitigated oxidative stress and neuroinflammation and improved learning and memory performance in an Aβ-induced mouse model of AD [9]. Mechanistically, treatment restored the protein levels of SLC7A11, GPX4, FTH1, and HO-1 while reducing those of ACSL4 and TFRC, bringing these six ferroptosis-related proteins close to control levels [129].

Taken together, current evidence suggests that miRNA-mediated ferroptosis regulation in AD converges mainly on dysregulated iron homeostasis, impairment of antioxidant defense, NOX4-associated oxidative stress, and glial–neuronal crosstalk. Further studies are needed to define cell-type-specific regulatory mechanisms, strengthen causal validation of ferroptosis dependency, identify direct downstream targets more precisely, and determine whether miRNA-based interventions can confer durable cognitive benefit in vivo.

## 4. Exosome-Based Therapeutics Targeting MicroRNA-Regulated Ferroptosis Pathways

Exosomes can protect miRNA cargo from nuclease degradation, facilitate intercellular transfer, and potentially enable delivery across the blood–brain barrier, thereby making them a promising platform for targeting miRNA-regulated ferroptosis pathways in neurological disorders [13]. Table 2 summarizes selected preclinical studies evaluating exosome-based therapeutic strategies targeting miRNA-regulated ferroptosis pathways in neurological disorders. Studies were included based on three criteria: use of an exosome-based therapeutic intervention, evaluation in preclinical models of neurological disorders, and identification of a defined miRNA regulatory axis involving ferroptosis-related targets.

## 5. Conclusions

Ferroptosis has emerged as an important mechanism of neuronal injury in a wide range of neurological disorders, and the evidence summarized in this review indicates that miRNAs constitute a critical upstream regulatory layer of this process. By targeting key nodes involved in antioxidant defense, lipid peroxidation, and iron metabolism, miRNAs influence ferroptotic susceptibility and thereby shape disease progression in ischemic stroke, intracerebral hemorrhage, epilepsy, toxic encephalopathy, spinal cord injury, Parkinson’s disease, and Alzheimer’s disease. Representative regulatory axes centered on SLC7A11/GPX4, Nrf2-related antioxidant signaling, ACSL4-dependent lipid remodeling, and iron-handling molecules such as FTH1, TfR1, FPN, and IRP2 collectively support a mechanistic link between post-transcriptional gene regulation and ferroptosis-associated neuronal damage.

At the same time, the current evidence remains largely preclinical, and several issues still limit translational progress, including incomplete validation of ferroptotic dependency, insufficient resolution of cell-type- and stage-specific miRNA effects, and persistent challenges in delivery, targeting specificity, and safety. Nevertheless, the convergence of mechanistic studies, biomarker discovery, and emerging exosome-based delivery strategies continues to strengthen the therapeutic relevance of this field. Future studies should focus on establishing the causal contribution of ferroptosis across defined cell types and disease stages, with particular attention to cell-type-specific miRNA regulatory networks. In parallel, advances in delivery efficiency, targeting precision, biosafety, and biomarker-guided patient stratification will be essential for translating miRNA-based anti-ferroptotic strategies into clinically viable interventions. Overall, miRNA-mediated modulation of ferroptosis represents a promising framework for understanding neuronal vulnerability and developing mechanism-based interventions in neurological disorders.

## Figures and Tables

**Table 1:** Key molecular regulators of ferroptosis and their regulatory microRNAs in neurological disorders.

Key Molecular Regulators of Ferroptosis	Ferroptosis-Related Process	Regulatory miRNAs	Related Neurological Disorders	References
GPX4	Lipid peroxide detoxification	miR-423-3p	Spinal cord injury	[18]
SLC7A11	Cystine import	miR-494, miR-378a-3p	Lead encephalopathy, Ischemic stroke	[19,20]
Nrf2	Antioxidant response	miR-128-3p	Spinal cord injury	[21]
FTH1	Iron sequestration	miR-21, miR-335	Arsenic encephalopathy, Parkinson’s disease	[22,23]
TfR1	Iron uptake	miR-125b-2-3p	Manganese encephalopathy	[24]
FPN	Iron efflux	miR-124	Intracerebral hemorrhage	[25]
FSP1	Antioxidant defense	miR-672-3p, miR-5627-5p	Spinal cord injury	[26,27]
ACSL4	Lipid peroxidation	miR-159a, miR-29a-3p	Ischemic stroke, Intracerebral hemorrhage	[28,29]

**Table 2:** Preclinical studies of exosome-based therapeutics targeting microRNA-regulated ferroptosis pathways in neurological disorders.

Exosome Source	Neurological Disorder	Key miRNA Involved	Ferroptosis-Related Target	Administration Method	References
Adipose-derived mesenchymal stem cells	Ischemic stroke	miR-760-3p	CHAC1	Intranasal administration	[74]
Umbilical cord-mesenchymal stem cells	Ischemic stroke	miR-494	SLC7A11	Intravenous injection	[19]
Adipose-derived mesenchymal stem cells	Intracerebral hemorrhage	miR-19b-3p	IRP2	Intravenous injection	[85]
Young healthy human plasma	Intracerebral hemorrhage	miR-25-3p	p53	Intracerebral ventricular injection	[84]
Bone marrow mesenchymal stem cells	Spinal cord injury	miR-219-5p	Nrf2	Intravenous injection	[113]
Mesenchymal stem cells	Spinal cord injury	miR-5627-5p	FSP1	Intravenous injection	[27]
Hypoxia-preconditioned adipose-derived stem cells	Spinal cord injury	miR-423-3p	GPX4	Intravenous injection	[18]

## Data Availability

No new data were created or analyzed in this study. Data sharing is not applicable to this article.
